# The effects of zoledronate on the survival and function of human osteoblast-like cells

**DOI:** 10.1186/s12891-015-0818-5

**Published:** 2015-11-16

**Authors:** Kuo-Chin Huang, Chin-Chang Cheng, Po-Yao Chuang, Tien-Yu Yang

**Affiliations:** Department of Orthopaedic Surgery, Chang Gung Memorial Hospital, Chiayi, Taiwan; Chang Gung University College of Medicine, Taoyuan, Taiwan; Present address: No. 6, West Section, Chia-Pu Road, Pu-Tz City Chiayi County, 61313 Taiwan

**Keywords:** Zoledronate, Bisphosphonates, Osteoblast, Bone regeneration, Bone repair

## Abstract

**Background:**

Prolonged bisphosphonate treatment might suppress bone remodeling to the extent that normal bone repair is impaired. While this adverse side effect is usually ascribed to the negative effects of bisphosphonates on osteoclast survival and function, these effects on osteoblasts are still unclear.

**Methods:**

In the current study, we hypothesized that zoledronate (ZOL) at the μM level might present negative effects on osteoblast survival and function. In vitro analyses of proliferation, migration and differentiation were performed on human osteoblast-like cells.

**Results:**

Our results revealed that ZOL treatment dose- and time-dependently induced apoptosis of osteoblasts after concentrations had reached 10 μM (*p* < 0.001). The concentrations at which ZOL inhibited osteoblast migration by 50 % were between 10 and 15 μM. Moreover, there was a dose-dependent reduction in the extent of matrix mineralization, but without a concomitant inhibition of osteogenic differentiation in terms of secreted type I collagen and osteocalcin and of alkaline phosphatase activity per viable cell. Analyses of the expression of osteogenic genes confirmed that ZOL at the μM level had no effects on osteogenic differentiation of osteoblasts.

**Conclusion:**

We concluded that ZOL at the μM level affected osteoblast survival and migration, but did not affect differentiation. The pathophysiological implications of ZOL at the μM level on skeletal disorders need to be investigated and clarified in the future researches.

**Electronic supplementary material:**

The online version of this article (doi:10.1186/s12891-015-0818-5) contains supplementary material, which is available to authorized users.

## Background

Bisphosphonates are the most widely used and effective agents against resorption of bone matrix by osteoclasts and have become an essential part of treatment in patients with established osteoporosis and in patients with risk factors for developing osteoporosis [1–3]. Like pyrophosphate, bisphosphonates bind strongly to the bone mineral [4] and are deposited in areas where minerals are exposed to body fluids, especially focal high-turnover bone lesions such as the microdamaged skeleton. Moreover, they are particularly resistant to enzymatic and chemical breakdown in vivo [3]. After focal deposition, they inhibit bone resorption through the mechanism of internalization by osteoclasts that interfere with various vital biochemical processes [1–6]. With such pharmacological properties that differentiate them from all other anti-osteoporosis agents, clinicians and researchers deduce that bisphosphonates might accumulate at the microdamaged skeletal lesions during long-term treatment that might lead to the persistence of their effects, both intended and unintended, even after treatment has been discontinued.

Clinical trials [7, 8] report that treatment with parenteral bisphosphonates, particularly zoledronate (ZOL), significantly decreases both the risk of osteoporotic fractures and the mortality after fragility hip fractures. While their main effects in vivo are on osteoclast survival and function, some controversial effects on osteoblasts have been reported; for example, that bisphosphonates at the nM-level protect osteoblasts from apoptosis [9, 10]. Evidence from human and animal studies [11–13] also suggests that prolonged treatment with bisphosphonates suppresses bone formation in vivo. These conflicting results could be a matter of cumulative-dose exposure secondary to long-term treatment (μM, not nM level) and should be investigated. We therefore hypothesized that ZOL at the μM level (1) inhibits osteoblast proliferation and induces osteoblast apoptosis, (2) inhibits osteoblast migration, and (3) inhibits osteogenic differentiation and matrix mineralization of osteoblasts.

## Methods

### Cell lines and cell cultures

We used the human osteoblast-like cell lines MG-63 (CRL-1427) and G-292 (CRL-1423) (American Type Culture Collection, Rockville, MD, USA), which are widely used in studies of osteoblast proliferation and differentiation. Both cell lines were maintained at 37 **°**C in a 5 % CO_2_ humidified incubator and their cell-specific culture media (minimal essential medium and McCoy’s 5A medium, respectively), supplemented with 10 % heat-inactivated fetal bovine serum (FBS), 100 U/ml of penicillin G, and 100 μg/ml of streptomycin, which were changed every 3 days. ZOL (Novartis Pharma, Basel, Switzerland) was dissolved in sterile ddH_2_O and used as stock solution at a concentration of 1.0 mg/ml. We serially diluted the stock solution with the cell-specific media so that the ZOL-conditioned media at different μM levels were prepared for experiments. Each experiment was repeated at least 3 times. MG-63 and G-292 cells were plated at a density of 6 × 10^5^ and 9 × 10^5^ cells per well in 6-well plates or at 5 × 10^3^ and 1 × 10^4^ cells per well in 96-well plates, respectively.

### Analyses of cell proliferation, apoptosis, and migration

Cell proliferation was analyzed using XTT assays (Biological Industries, Kibbutz Beit-Haemek, Israel), for which the cells were plated in 96-well plates and allowed to attach for 24 h. The culture media were then changed to the ZOL-conditioned and control media for a 48-h treatment. An ELISA plate reader (Thermo Labsystems Multiskan RC, Vantaa, Finland) was used to measure the absorbance of the samples at a wavelength of 490 nm.

Apoptosis was detected using chromatin condensation and fragmentation with H342 dye (Sigma-Aldrich, St. Louis, MO, USA). Cells were seeded in 6-well plates, grown for 24 h, and then incubated with the ZOL-conditioned and control media for 48 and 72 h. H342 dye from the stock solution, originally suspended in 1 mM of ddH2O, was added to the cell suspension for a total of 10 μM. The cells were then incubated for 60 min, and the apoptotic cells were examined under a fluorescence microscope.

We studied the effects of ZOL at the μM level on osteoblast recruitment using a cell migration assay. Cells were seeded in 6-well plates, allowed to attach for 24 h, and then incubated with the ZOL-conditioned and control media for 48 h. After they had been treated, a pipette tip was used to make an I-shaped scratch on the well. The scratch was followed for its closure and photographs were taken under a microscope every 12 h for 3 days.

### Analyses of cell differentiation and matrix mineralization

We used Alizarin Red-S (ARS) staining to study the effects of ZOL on matrix mineralization. After they had been incubated with the ZOL-conditioned and control media for 7 days, the cells were washed with PBS, and then fixed in ice-cold 70 % ethanol for at least 1 h. The ethanol was then removed and the cells were stained with 40 mM ARS (Sigma-Aldrich) (pH 4.2), for 10 min at room temperature. The stained cells were then photographed under a microscope.

Cell differentiation was analyzed using ELISA test kits (Takara Bio, Otsu, Shiga, Japan; and Invitrogen, Carlsbad, CA, USA) that were enzyme immunoassays (EIA) designed to determine type I collagen (COL-1) and osteocalcin (OCN) directly in biological fluids, which were the culture supernatants in this case. Samples were processed and placed in an ELISA plate reader to determine the absorbance at 450 nm against 690 nm (as a reference). Alkaline phosphatase (ALP) activity was also quantified (SensoLyte pNPP ALP Assay Kit; AnaSpec, San Jose, CA, USA). After they had been treated with the working solution, the amount of ALP product (*p*-nitrophenolate) released by the reaction was measured using a spectrophotometer at 405 nm. All values were normalized against the numbers of cells in the sample.

### Analyses of the expression of osteogenic genes

Total RNA was isolated from the cells that were treated as described above after expansion with normal media or STEMPRO Osteogenesis Differentiation Medium (Invitrogen) for 72 h (Total RNA Isolation Kit; Life Technologies, Gaithersburg, MD, USA). We assessed the total RNA samples for quality control before using a PCR assay (Human Osteogenesis RT^2^ Profiler PCR Array; SABiosciences [formerly SuperArray Bioscience], Frederick, MD, USA). Samples were screened for the expression of 84 genes implicated in differentiation and bone metabolism. A gene was regarded as constitutively expressed if it was detected at a cycle threshold (CT) of ≤ 35. Genes with CT values > 35 were considered as not expressed. Fold-change and fold-regulation of each gene were calculated as the difference in gene expression between the ZOL-treated and vehicle-treated osteoblasts.

### Statistical analysis

SPSS 12.0.1 (SPSS Inc., Chicago, IL, USA) was used for all analyses: one-way analysis of variance (ANOVA) and then a Dunnett’s post-hoc for the differences between the means of the experimental and control groups. Quantitative data are means ± standard deviation (SD). Significance was set at *p* < 0.05 (two-tailed).

## Results

### Effects of ZOL on cell proliferation, apoptosis, and migration of osteoblasts

**Fig. 1:**
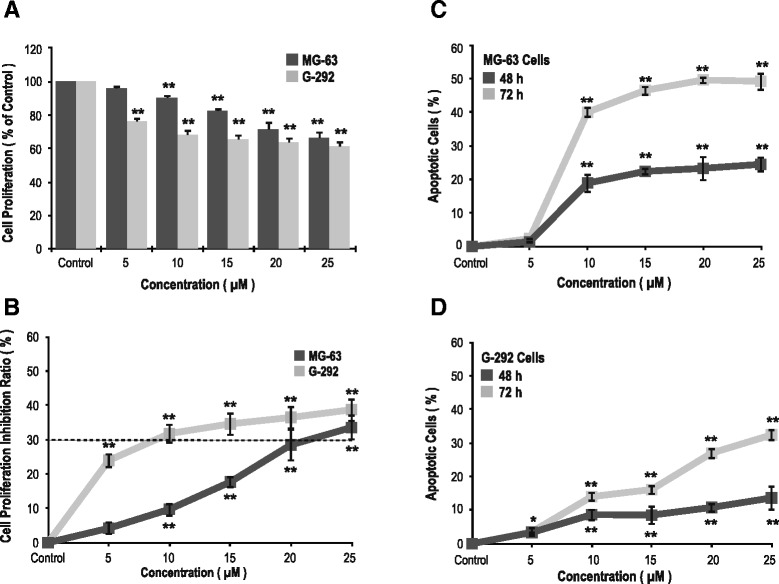
Zoledronate (ZOL) at the μM level presents a dose-dependently inhibitory effect on cellular proliferation of MG-63 and G-292 cells by inducing apoptosis. Sequential changes of (**a**) cell proliferation and (**b**) cell proliferation inhibition ratio of MG-63 and G-292 cells treated with ZOL for 48 h. Sequential changes of apoptotic cells of (**c**) MG-63 cells and (**d**) G-292 cells treated with ZOL for 48 h and 72 h. Data are mean ± standard deviation (SD). *N* = 3–5 for each experiment; *p < 0.05, **p < 0.001 (vs. the control group; ANOVA)

**Fig. 2:**
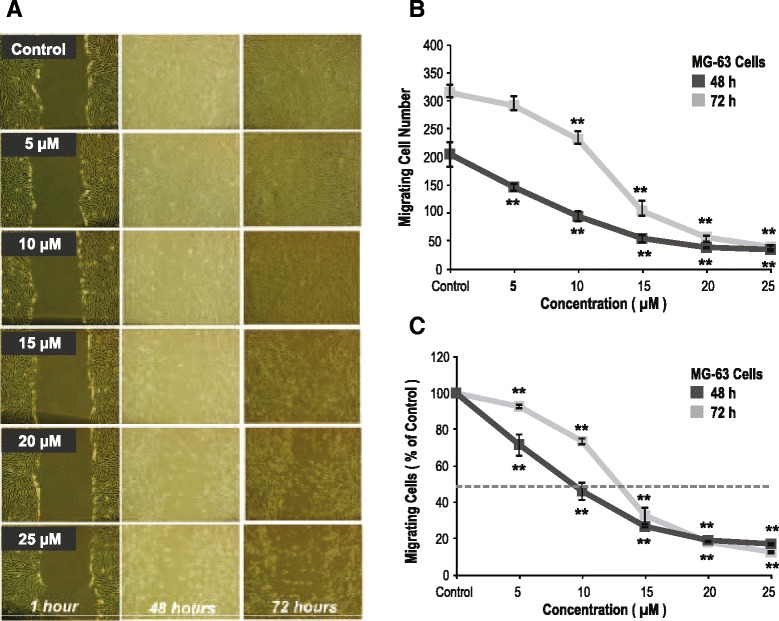
Zoledronate (ZOL) at the μM level presents a dose-dependently inhibitory effect on cellular migration of MG-63 cells. **a** Photographs of MG-63 cell migration assay. Sequential changes of (**b**) absolute and (**c**) relative numbers of migrating MG-63 cells treated with ZOL. Data are mean ± standard deviation (SD). *N* = 3–5 for each experiment; *p < 0.05, **p < 0.001 (vs. the control group; ANOVA)

After a 48-h ZOL treatment, the osteoblast proliferation rate was dose-dependently downregulated (Fig. 1a). There were significant differences between the ZOL-treated and vehicle-treated osteoblasts, with *p* < 0.001 at 10 and 5 μM and above for MG-63and G-292 cells, respectively. After a 48-h ZOL treatment, a reduction of more than 30 % was observed while the concentration reaching the level of 20 μM (Fig. 1b). Besides, ZOL treatment stimulated apoptosis in a dose- and time-dependent manner at 10 μM and above (*p* < 0.001) (Fig. 1c, d). A similar pattern of the effects of ZOL at the μM level was observed in rat stromal cells (R7500). (Additional file 1: Figure S1) Cell migration assays of MG-63 cells reveal that ZOL treatment decreased the number of cells that migrated into the scratch in a dose-dependent manner (Fig. 2a, b). The concentrations at which ZOL inhibited migration by 50 % were between 10 and 15 μM (Fig. 2c).

### Effects of ZOL on cell differentiation and matrix mineralization of osteoblasts

**Fig. 3:**
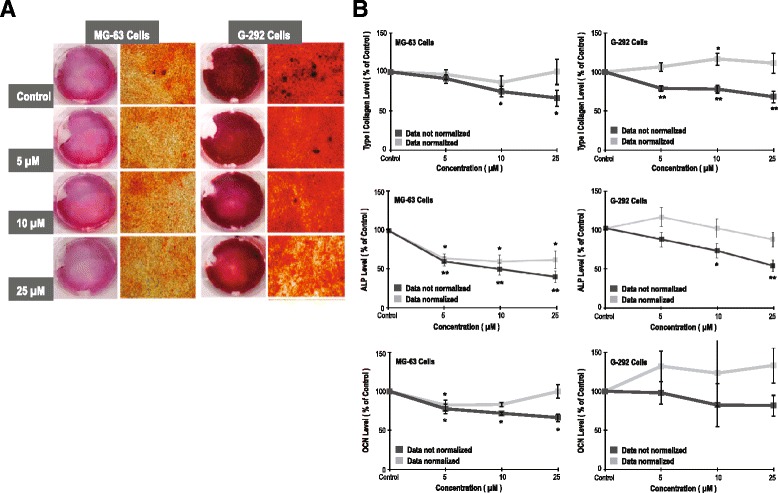
Zoledronate (ZOL) at the μM level presents a negative effect on matrix mineralization of MG-63 and G-292 cells. **a** Photographs of Alizarin red staining of differentiating MG-63 and G-292 cells on the 7th day showed a negative association between ZOL concentrations and the amount of bone nodule formation. **b** Sequential changes of the levels of type I collagen, alkaline phosphatase (ALP) and osteocalcin (OCN) of MG-63 and G-292 cells treated with ZOL. Data are mean ± standard deviation (SD). *N* = 3–5 for each experiment; *p < 0.05, **p < 0.001 (vs. the control group; ANOVA)

ZOL treatment for 7 days dose-dependently inhibited bone nodule formation (Fig. 3a). A similar pattern of the effects of ZOL at the μM level was observed in rat stromal cells (R7500) again. (Additional file 2: Figure S2) To clarify the effects of ZOL on osteoblast differentiation and matrix mineralization, we assessed COL-1 and OCN secretion and ALP activity. ZOL treatment dose-dependently inhibited COL-1 and OCN secretion. However, there were no significant differences in either COL-1 or OCN secretion per viable cell between the ZOL-treated and vehicle-treated osteoblasts. Meanwhile, there was a dose-dependent decrease of total ALP activity and that per viable cell (Fig. 3b).

### Effects of ZOL on the expression of osteogenic genes

**Fig. 4:**
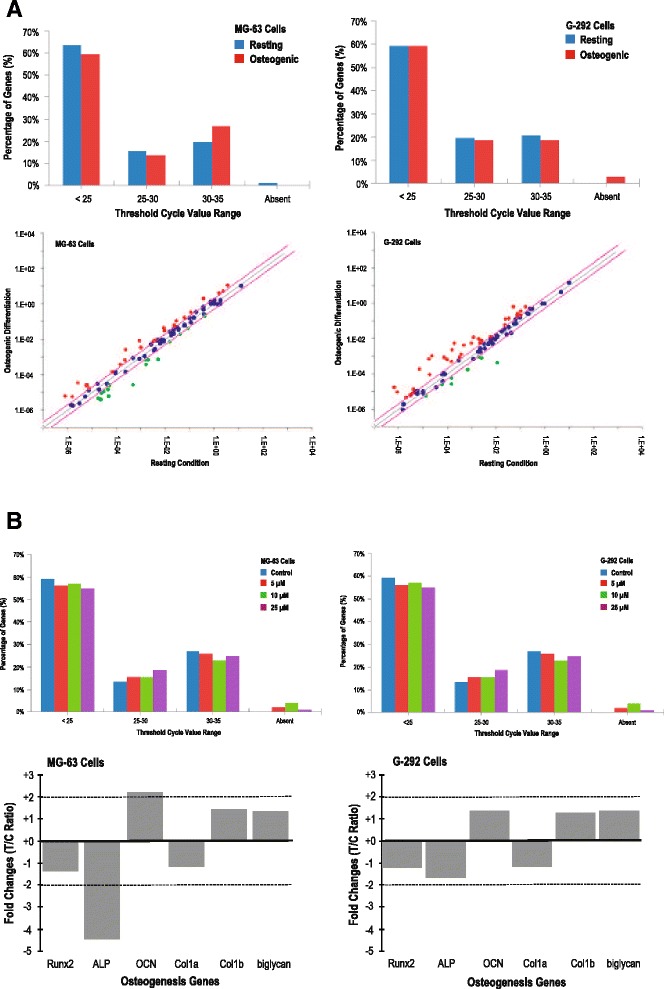
Zoledronate (ZOL) at the μM level does not alter the osteogenic gene expression. **a** Fold changes of osteogenic gene expression in MG-63 and G-292 cells cultured in normal and osteogenic differentiation media. **b** Effects of ZOL at the μM level on the fold-regulation of osteogenic gene expression in MG-63 and G-292 cells post osteogenic induction

The distribution patterns of constitutive genes were similar, but after osteogenic induction, gene expression levels increased for both cell types (Fig. 4a). In total, 25 genes for MG-63 cells, and 33 for G-292 cells, showed a treated/control (T/C) ratio of > +2 in response to osteogenic induction. Of these, Runx2 expression levels increased up to 2.4 times for MG-63 cells, and 5.7 times for G-292 cells. Additionally, expressions of COL-1 (Col-1α1 and Col-1α2) and bone matrix proteins (ALP, OCN, and biglycan) were all upregulated in both cell types. We also investigated the effect of ZOL on the osteogenic potential of osteoblasts after osteogenic induction. ZOL at different levels did not change the patterns of constitutive gene expression (Fig. 4b). There was no change in the fold-regulation (T/C ratio > ± 2) for Runx2, Msx1, NFκB, the SMAD family (SMAD 1–4), and the TGF/BMP superfamily expression. After ZOL treatment, the expression levels of ALP were significantly lower: a 4.3-fold change for MG-63 cells and a 2.2-fold change for G-292 cells; however, the mRNA levels of OCN, Col-1α1, Col-1α2 and biglycan were all unchanged.

## Discussion

We found that ZOL at the μM level had a dose-dependently negative effect on cell proliferation, survival, migration, and matrix mineralization of both the MG-63 and the G-292 human osteoblast-like cell lines. There was, however, no significant difference in the expression and production of osteogenic differentiation markers per viable cell between the ZOL-treated and vehicle-treated osteoblasts. The diminished extent of matrix mineralization after ZOL treatment was, therefore, ascribable to decreased cell proliferation and increased apoptosis, not to decreased osteogenic differentiation of osteoblasts. Our findings also provide a clue to the conflicting effects of bisphosphonates on the survival and function of human osteoblasts [9–13]. Although bisphosphonates at the nM level might have an anabolic effect on bone [9, 10], bisphosphonates at the μM level had a negative effect on the survival and function of osteoblasts.

Bisphosphonates have specific pharmacological properties; the absence of decay, possible dose-accumulation, and prolonged retention in focal high-turnover bone lesions such as those in microdamaged skeletons [1–3]. This may lead to the persistence of their effects on bone tissue, both intended and unintended, even after discontinuation of treatment [1]. Although widely used in clinical practice [7, 8], prolonged bisphosphonate treatment might finally impair bone repair and predispose bones to atypical fractures [11]. The actual concentration levels of bisphosphonates that osteoblasts in the matrix microenvironment are exposed to under pharmacological conditions remain unclear; however, an in vivo study [14] using a fluorescent bisphosphonate analogue, far-red fluorescent pamidronate, reported that the bone uptake of bisphosphonates is linear with parentally administered doses.

An increasing body of evidence suggests that bisphosphonates accumulate in the bone matrix after repeated dosing and might blunt the anabolic response of parathyroid hormone [14–19]. Clinically, the cumulative dose effects on bone formation might contribute to osteonecrosis of the jaw in patients treated with a high-frequency dosing regimen [15] and to atypical fractures in patients with osteoporosis and a prolonged dosing regimen [11, 12, 16]. Drug-induced unrepaired microdamage in focal high-turnover bone lesions such as the mandible and proximal femur is thought to be the cause [14, 20, 21]. These lesions might uptake bisphosphonates up to the μM level in the resorption space even after only a single dose [14, 17]. By using the reported peak local concentrations in the resorption space, we confirmed that ZOL at the μM level had a negative effect on the survival and function of human osteoblasts.

This study has some limitations. First, it was in vitro and its findings cannot necessarily be generalized to include in vivo effects. Second, the human osteoblast-like cell lines used were derived from osteosarcoma because of their ability to grow for long periods in culture. Third, the aim of the study was to identify the effects of ZOL at the μM level on the survival and function of osteoblasts but not to investigate mechanisms of interaction. Randomized prospective controlled clinical trials and in vitro studies on the regulation mechanisms and signaling pathways are needed.

## Conclusion

ZOL at the μM level might negatively affect bone formation by directly inhibiting proliferation, survival, and migration of osteoblasts and then indirectly inhibiting matrix mineralization. This finding raises the possibility that atypical fractures might in part be caused by the negative effects on osteoblasts and, therefore, the unrepaired microdamage of cortical bone in high-turnover bone lesions in, for example, the subtrochanteric and diaphyseal femur. Further studies are needed to investigate and clarify the pathophysiological implications of ZOL at the μM level on related skeletal disorders.

## Additional files

Additional file 1:
**Figure S1.** Zoledronate (ZOL) at the μM level presents a dose-dependently inhibitory effect on cellular proliferation of rat stromal cells (R7500). (EPS 825 kb) Additional file 2:
**Figure S2.** Zoledronate (ZOL) at the μM level presents a negative effect on matrix mineralization of rat stromal cells (R7500). (EPS 1961 kb)
